# The Development and Validation of a Predictive Model for Voriconazole-Related Liver Injury in Hospitalized Patients in China

**DOI:** 10.3390/jcm12134254

**Published:** 2023-06-25

**Authors:** Guirong Xiao, Yiyao Liu, Yanhua Chen, Zhiyao He, Yan Wen, Ming Hu

**Affiliations:** 1 Department of Pharmacy, West China Hospital, Sichuan University, Chengdu 610041, China; xgr1985@scu.edu.cn (G.X.);; 2 West China School of Pharmacy, Sichuan University, Chengdu 610041, China; 3 West China Biomedical Big Data Center, West China Hospital, Sichuan University, Chengdu 610041, China; 4 Department of Pharmacy, Hospital of Chengdu University of Traditional Chinese Medicine, Chengdu 610072, China

**Keywords:** voriconazole, liver injury, disposal cost, risk factor, prediction model, nomogram

## Abstract

Voriconazole is widely used in the treatment and prevention of invasive fungal diseases. Common drug-induced liver injuries increase the economic burdens and the risks of premature drug withdrawal and disease recurrence. This study estimated the disposal cost of voriconazole-related liver injury, explored the risk factors of voriconazole-related liver injury in hospitalized patients, and established a predictive model of liver injury to assist clinicians and pharmacists in estimating the probability or risk of liver injury after voriconazole administration to allow for early identification and intervention in patients at high risk of liver injury. A retrospective study was conducted on the selected inpatients whose blood concentration of voriconazole was measured in the West China Hospital of Sichuan University from September 2016 to June 2020. The incidence and disposal cost of voriconazole-related liver injuries were calculated. The incidence of voriconazole-related liver injury was 15.82% (217/1372). The disposal cost has been converted to 2023 at a discount rate of 5%. The median (P_25_, P_75_) disposal cost of severe liver injury (*n* = 42), general liver injury *(n* = 175), and non-liver injury (*n* = 1155) was 993.59 (361.70, 1451.76) Chinese yuan, 0.00 (0.00, 410.48) yuan, and 0.00 (0.00, 0.00) yuan, respectively, with a statistically significant difference (*p* < 0.001). Single factor analysis and multiple factor logistic regression were used to analyze the risk factors of voriconazole-related liver injury. The voriconazole-related liver injury was related to the trough concentration (*C*_min_, OR 1.099, 95% CI 1.058–1.140), hypoproteinemia (OR 1.723, 95% CI 1.126–2.636), and transplantation status (OR 0.555, 95% CI 0.325–0.948). The prediction model of liver injury was Logit (P)= −2.219 + 0.094 × *C*_min_ + 0.544 × *H_ydroproteinemia_* − 0.589 × *Transplantation*, and the prediction model nomogram was established. The model validation results showed that the C-index of the derivation set and validation set was 0.706 and 0.733, respectively. The area under the curve (AUC) of the receiver operating characteristic (ROC) curve was 0.705 and 0.733, respectively, indicating that the model had good prediction ability. The prediction model will be helpful to develop clinical individualized medication of voriconazole and to identify and intervene in the cases of patients at high risk of voriconazole-related liver injury early on, in order to reduce the incidence of voriconazole-related liver injuries and the cost of treatment.

## 1. Introduction

Voriconazole is widely recommended as the first choice for the treatment of aspergillosis by many guidelines, and is increasingly used for fungal diseases caused by cryptococcus and fluconazole-resistant candida [1,2,3,4]. Common adverse reactions to voriconazole include abnormal liver biochemical indicators and liver injury. A global multicenter prospective study reported that the incidence of abnormal liver biochemical indicators in the voriconazole group was 12.2% (35/287, median treatment course 64 days) [5]. A meta-analysis of voriconazole in the treatment of chronic pulmonary aspergillosis showed that the incidence of voriconazole-related liver injury was 5.5% (*n* = 366, eight studies) [6]. The UK Medicines and Healthcare Products Regulatory Agency (MHRA) has warned about the risk of voriconazole-related liver injury. The Beijing ADR Monitoring Center of China collected 515 cases of voriconazole-related adverse reactions from medical institutions from January 2011 to June 2021. The top three adverse reactions were hallucination, liver injury, and visual impairment [7]. Adverse drug reactions lead to premature drug withdrawal and increase the risk of disease recurrence. Meta-analysis showed that 12.8% (47/366) of patients stopped taking medicine due to voriconazole-related adverse reactions [6]. Bogler Y. et al. reported that 22.9% (48/210) of patients with hematopoietic stem cell transplantation (HCT) stopped taking medicine prematurely due to voriconazole-related liver injury [8]. Bongomin F. et al. found in their retrospective study on chronic pulmonary aspergillosis that 42.2% (43/102) of the patients who stopped early mainly due to adverse reactions had relapsed within six months after drug withdrawal [9].

There have been some studies on the risk factors of voriconazole-related liver injury, and many of those studies have shown that voriconazole-related liver injury is related to serum trough concentration [1,2,10], while few suggested that voriconazole-related liver injury may be related to *CYP2C19* gene polymorphism or not [11,12], but not to *UGT1A4* gene polymorphism, age, sex, body mass, and body mass index. There is no prediction model available between voriconazole-related liver injury and multiple factors. Only two quantitative studies on the relationship between liver injury or abnormal liver biochemical indicators and a single factor (blood concentration) have been reported. In 2006, Tan K. et al. predicted that the probability of abnormal aspartate aminotransferase (AST), alkaline phosphatase (ALP), and bilirubin would increase by 13.1%, 16.5% and 17.2%, respectively, when the serum trough concentration of voriconazole increased by 1 mg/L [13]. In 2021, Hanai Y. et al. established the relationship between liver injury (*y*) and serum trough concentration (*x*, range 1–6 mg/L) *y* = 0.1198 × e^0.2298*x*^ (*p* = 0.007) [14].

Integrating multiple risk factors and developing a risk prediction model can help doctors or pharmacists evaluate the probability or risk of adverse reactions after drug use, so as to make wise clinical decisions. The application of risk prediction models in adverse drug reactions is still rare. This study focuses on the common liver injury that often needs to be detected by laboratory examination, looks for the risk factors, and establishes a risk prediction model for voriconazole-related liver injury based on the real world data of medical institutions, to allow for early identification and intervention in patients at high risk of liver injury and to promote adequate duration of medication.

## 2. Materials and Methods

### 2.1. Study Design and Patient Selection

This retrospective study was performed on a group of hospitalized patients in the West China Hospital of Sichuan University from 1 September 2016 to 30 June 2020. Patient’s basic information, diagnosis, medical order sheet, and laboratory tests (liver biochemical indicators, voriconazole blood concentration) were automatically extracted through the hospital information system. After data cleaning, patients with abnormal liver biochemical indicators were manually checked in the electronic medical records to determine whether voriconazole was related, and the disposal cost was analyzed according to the medical order and the cost list. inclusion criteria were that hospitalized patients who received voriconazole and therapeutic drug monitoring (TDM) were eligible. Exclusion criteria were: (1) age ≤ 14 (the pharmacokinetic characteristics and dosage of voriconazole in children under 14 years old are different from those in adults); (2) duration of voriconazole usage < 5 d; (3) not steady-state trough concentration; (4) within 5 days prior to voriconazole use, the liver biochemical indicators were not measured or were higher than the upper limit; (5) during the use of voriconazole, the liver biochemistry was not measured within ±3 days of the TDM time, or the liver biochemical changes were caused by other factors (diseases, other drugs). Those meeting at least one of the above items were excluded.

Repeated hospitalization of the same patient was accounted for per the number of hospitalizations. Only one blood–drug concentration result was selected in each hospitalization if there were multiple blood–drug concentration results. If the dosage of voriconazole (excluding load dose) did not change, the last blood–drug concentration result was selected; if the dosage of voriconazole changed, we found out the corresponding time period of the first blood–drug concentration under the same dosage, and then selected the last blood–drug concentration result within the time period. Drug dosage was considered unchanged as long as the same amount of drug was administered, regardless of difference in administration routes, dosage forms, specifications, and manufacturers.

**Figure 1:**
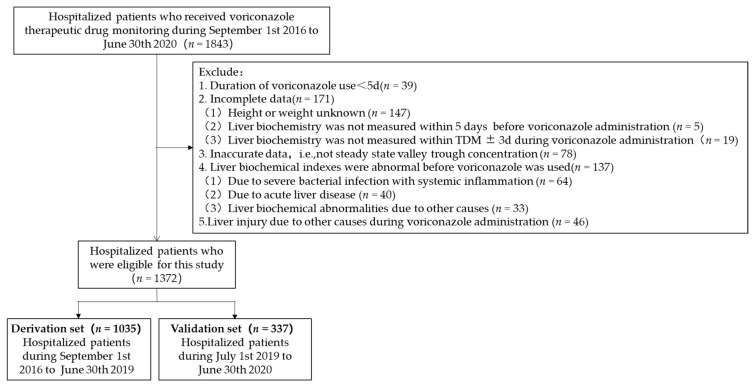
Flowchart of patients included in this study.

The cases that met the inclusion and exclusion criteria were divided into the derivation set and the verification set according to their admission time. The derivation set included cases from 1 September 2016 to 30 June 2019 (*n* = 1035), and the verification set included cases from 1 July 2019 to 30 June 2020 (*n* = 337). The predictive model was established in the derivation set and independently validated in the validation set. Details about patients’ selection are shown in Figure 1.

### 2.2. Variable Definitions

Patients’ basic information (age, gender, height, weight, body mass), fungal disease type, concomitant diseases, voriconazole dosage, blood concentration monitoring results, and liver biochemical indicators were collected. Concomitant diseases such as hypoproteinemia and transplantation status were judged according to clinical diagnosis. Liver biochemical indicators included alanine aminotransferase (ALT), AST, ALP, total bilirubin (TBil), and direct bilirubin (DBil).

Steady-state trough concentration: the patient’s administration time and TDM sampling time were extracted through the information system. Then, after 48 h of administration, a blood sample was taken within 2 h before the next administration and used to determine the steady-state trough concentration [15,16]. Domestic and international guidelines recommend monitoring the steady-state trough concentration of voriconazole, and the target trough concentration range is mainly 1.0–5.5 mg/L [1,2,17]. In this study, a trough concentration <1.0 mg/L was considered as low concentration, 1.0–5.5 mg/L was considered as standard, and >5.5 mg/L was considered as high concentration.

Abnormal liver biochemical indicators: Any laboratory indicator (ALT, AST, ALP, TBil, DBil) that was greater than the upper limit of normal (ULN).

Drug-induced liver injury: There are many diagnostic criteria for drug-induced liver injury, mainly including the diagnostic criteria formulated by the Council for International Organizations of Medical Sciences (CIOMS) in 1990 [18], the diagnostic criteria published by the Drug-Induced Liver Injury Network (DILIN) in 2009 [19], and the diagnostic criteria formulated by the International DILI Expert Working Group (DEWG) in 2011 [20]. There are differences in the incidence of liver injury reported according to different diagnostic standards. Tan et al. compared the three standards in 2020, among 42,176 inpatients, 1707, 926, and 888 patients with drug-induced liver injury were diagnosed, respectively, using CIOMS, DILIN, and DEWG standards [21]. It can be seen that the CIOMS standard is more sensitive and can avoid missing cases. The DEWG and DILIN standards are more specific, facilitating the diagnosis of drug-induced liver injury. For the early detection and intervention of suspected cases of liver injury, the CIOMS standard with higher sensitivity was selected as the judgment standard of voriconazole-related liver injury in this study. CIOMS standard: (1) ALT > 2 × ULN; (2) DBil >2 × ULN; (3) AST or ALP or TBil > ULN, at least one of which > 2 × ULN. If one of the above three items is met, it will be judged as drug-induced liver injury [18].

Incidence of voriconazole-related liver injury: Number of new cases of voriconazole-related liver injury, or the number of total cases with voriconazole × 100%. The study investigated the frequency of new cases of liver injury during voriconazole treatment in hospitalized patients who met the inclusion and exclusion criteria.

Severity of adverse reactions: The General Adverse Event Terminology standard 5.0 of the National Cancer Institute was used as reference to determine the severity of liver injury by laboratory indicators (ALT, AST, ALP, TBil). ALT: 1–3 ULN = 1, 3–5 ULN = 2, 5–20 ULN = 3, >20 ULN = 4, AST: 1–3 ULN = 1, 3–5 ULN = 2, 5–20 ULN = 3, >20 ULN = 4, ALP: 1–2.5 ULN = 1, 2.5–5 ULN = 2, 5–20 ULN = 3, >20 ULN = 4, TBil: 1–1.5 ULN = 1, 1.5–3 ULN = 2, 3–10 ULN = 3, >10 ULN = 4 [22]. According to the grading results of the four indicators, the single indicator with the highest grading reflected the severity of liver injury. Grade ≥ 3 was severe liver injury, Grade 1–2 was general liver injury.

Cost: The disposal cost of adverse reactions (liver injury) in the direct medical cost, including the cost of hepatoprotective drugs, solvents, and infusion sets, excluding the increased examination cost due to adverse reactions, the cost of medical staff time, and the labor loss caused by patients’ prolonged hospitalization. The cost data was obtained from the medical order and the cost list in the electronic medical record, prior to Medicare reimbursement. Referring to the Chinese guidelines for pharmacoeconomic evaluations [23], the disposal cost was analyzed using a discount rate of 5% per year because the time span of the cases(from September 2016 to June 2020) was more than 1 year, and the cost was converted to the value equivalent in 2023. A sensitivity analysis of the discount rate between 0 and 8% was performed.

### 2.3. Statistical Methods

Statistical software IBM SPSS Statistics version 25 (IBM Corporation) and R version 4.1.2 (www.r-project.org) (accessed on 30 March 2023). were used for statistical analysis and model construction. The χ^2^ Test or Fisher’s Exact Test were used for counting data. If the measurement data conformed to the normal distribution, the mean ± standard deviation was used to express the measurement data and the independent sample *t*-test was used for the inter-group comparison. If the measurement data did not conform to the normal distribution, the [M (P_25_, P_75_)] was used and the Mann–Whitney U-rank sum test was used for the inter-group comparison. The disposal cost of adverse reactions did not conform to the normal distribution, and the Kruskal–Wallis H test was used for comparison among groups. Statistical significance was set at *p* ≤ 0.05.

In the derivation set, a total of 26 variables were screened via univariate analysis. If *p* < 0.1, this variable would progress to multivariable logistic regression. Additionally, independent predictors were determined when *p* < 0.05 in multivariable regression. Then, they were integrated to be an initial risk prediction model. This initial model was presented as a mathematical formula:

Logit(P) = ß_0_ + ß_1_ × *X*_1_ + ß_2_ × *X*_2_ + … + ß_n_ × *X*_n_

P represents the probability of liver injury occurrence. *X*_1_, *X*_2_, … *X*_n_ represent predictive variables we have selected. ß_1_, ß_2_, … ß_n_ refer to the regression coefficients of corresponding variables.

The R version 4.1.2 was used to construct the nomogram of the voriconazole-related liver injury prediction model. The receiver operating characteristic curve (ROC) was used to evaluate the effectiveness of the prediction model. The area under the curve (AUC) was calculated. The larger the AUC value, the higher the prediction value, and when AUC > 0.7, the results were statistically significant. The critical value of blood–drug concentration was calculated by Yoden’s index (Yoden’s index = sensitivity + specificity − 1).

## 3. Results

### 3.1. Clinical Characteristics of Patients

Among all cases (*n* = 1372), age ranged from 15 to 94 years old, males accounted for 68.44%, and Aspergillus accounted for 39.29%. Common complications included bacterial infection (35.86%), hypertension (35.50%), hypoproteinemia (24.78%), anemia (23.83%), diabetes (22.89%), malignant tumor (17.93%), chronic renal failure (15.52%), heart disease (12.10%), and kidney transplantation status (10.06%). The median (P25, P75) trough concentration of voriconazole was 2.66 (1.20, 5.00) mg/L, accounting for 58.09% in the target range (1.0–5.5 mg/L). There were differences between the two groups in the type of fungal disease, some concomitant diseases, the steady-state trough concentration of voriconazole, and liver biochemical indicators (*p* < 0.05) (Table 1).

### 3.2. Incidence of Voriconazole-Related Liver Injury

In all cases, the incidence of abnormal voriconazole-related liver biochemical indicators was 39.94% (548/1372), and the incidence of voriconazole-related liver injury was 15.82% (217/1372). The incidence of liver injury in the low voriconazole concentration group (<1.0 mg/L) was 2.87% (8/279), the incidence of liver injury in the standard group (1.0–5.5 mg/L) was 14.05% (112/797), and the incidence of liver injury in the high concentration group (>5.5 mg/L) was 32.77% (97/296). The incidence of liver injury increased with the increase in concentration (linear by linear association, Z = 97.269, *p* < 0.001).

### 3.3. Disposal Cost of Voriconazole-Related Liver Injury

The disposal cost has been converted to 2023 at a discount rate of 5%. The disposal cost of each group showed abnormal distribution (Table 2). The median (P_25_, P_75_) disposal cost of the non-liver injury group (including abnormal liver biochemical indicators) was 0.00 (0.00, 0.00) Chinese yuan, and the median disposal cost of the liver injury group was 101.90 (0.00, 786.48) yuan, with a statistically significant difference (Mann–Whitney U test, Z = −18.401, *p* < 0.001). The median disposal cost of the general liver injury group was 0.00 (0.00, 410.48) yuan, and the median disposal cost of the severe liver injury group was 993.59 (361.70, 1451.76) yuan, with a statistically significant difference (Mann–Whitney U test, Z = −6.371, *p* < 0.001). The Kruskal–Wallis H test was used to compare the disposal cost of non-liver injury, general liver injury, and severe liver injury. It also showed that with the aggravation of adverse reactions, the disposal cost increased (H = 418.794, *p* < 0.001). There was a statistically significant difference between the two (Bonferroni, adjusted *p* < 0.001). Spearman was used to analyze the correlation between the disposal cost and the degree of liver injury (assignment: non-liver injury = 1, general liver injury = 2, severe liver injury = 3), with a moderate-intensity correlation (rs = 0.512, *p* < 0.001).

A sensitivity analysis of the discount rate between 0 and 8% was performed (Table 3). The disposal cost of voriconazole-related liver injury was expressed as a mean value of 466.29~693.05 Chinese yuan and a median value of 79.84~117.31 yuan. In particular, the disposal cost of severe voriconazole-related liver injury increased to 853.02~1243.09 yuan on mean and 770.00~1128.31 yuan on median.

### 3.4. Predictors of Voriconazole-Related Liver Injury

In the derivation set, the voriconazole-related liver injury group included 152 patients, and the non-voriconazole-related liver injury group included 883 patients. Seven potential predictors with *p* < 0.1 were selected by univariate analysis, including *C*_min_, hypoproteinemia, transplantation, cryptococcosis, decompensated cirrhosis, heart disease, and anemia (Table 4).

According to multivariable logistic regression, four variables were eliminated, included cryptococcosis, decompensated cirrhosis, heart disease, and anemia (*p* > 0.05). Three factors were found to be independently associated with the voriconazole-related liver injury outcome, including *C*_min_ (OR = 1.099, 95% CI: 1.058–1.140, *p* < 0.001), hypoproteinemia (OR 1.723, 95%CI 1.126–2.636, *p* = 0.012), and transplantation (OR 0.555, 95%CI 0.325–0.948, *p* = 0.031) (Table 5).

The risk prediction model was exhibited as following:

Logit(P)= −2.219 + 0.094 × *C*_min_ + 0.544 × *Hypoproteinemia* − 0.589 × *Transplantation*

### 3.5. Development and Validation of Nomogram

**Figure 2:**
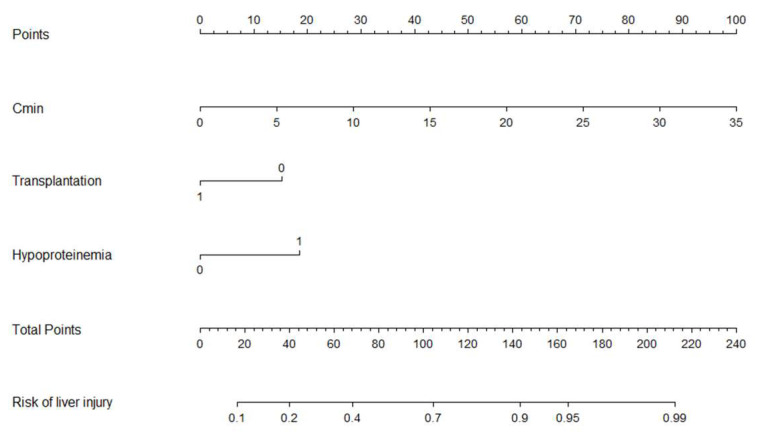
Nomogram for the prediction of voriconazole-related liver injury.

R software was used to draw the nomogram of the prediction model, as shown in Figure 2. There were six lines in the nomogram. The first line was the point distribution of the predictive variables, and the second to fourth lines were the predictive variables of liver injury (concentration is a continuous variable, and hypoalbuminemia and transplantation status are classified variables). The probability of occurrence of voriconazole-related liver injury was predicted by matching the sum of the total scores (the fifth line) with the scores on the total score table (the sixth line).

**Figure 3:**
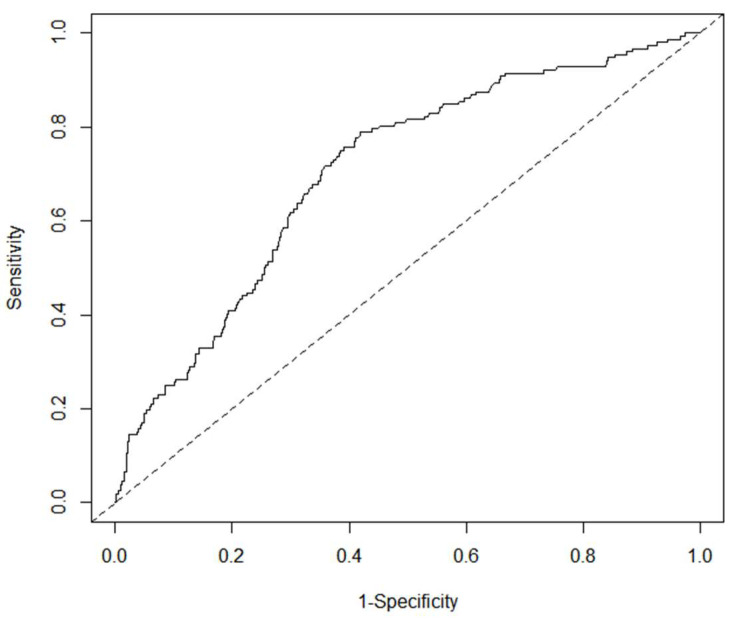

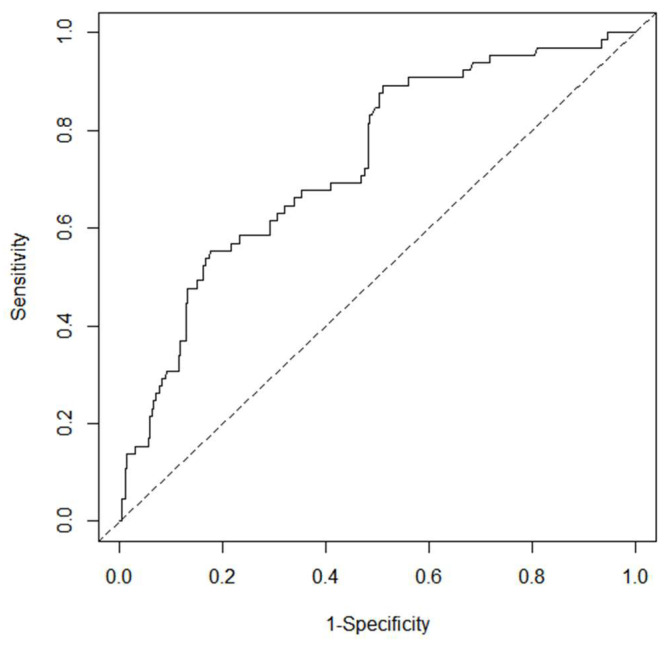
ROC curve of nomogram (above: derivation set, below: verification set).

**Figure 4:**
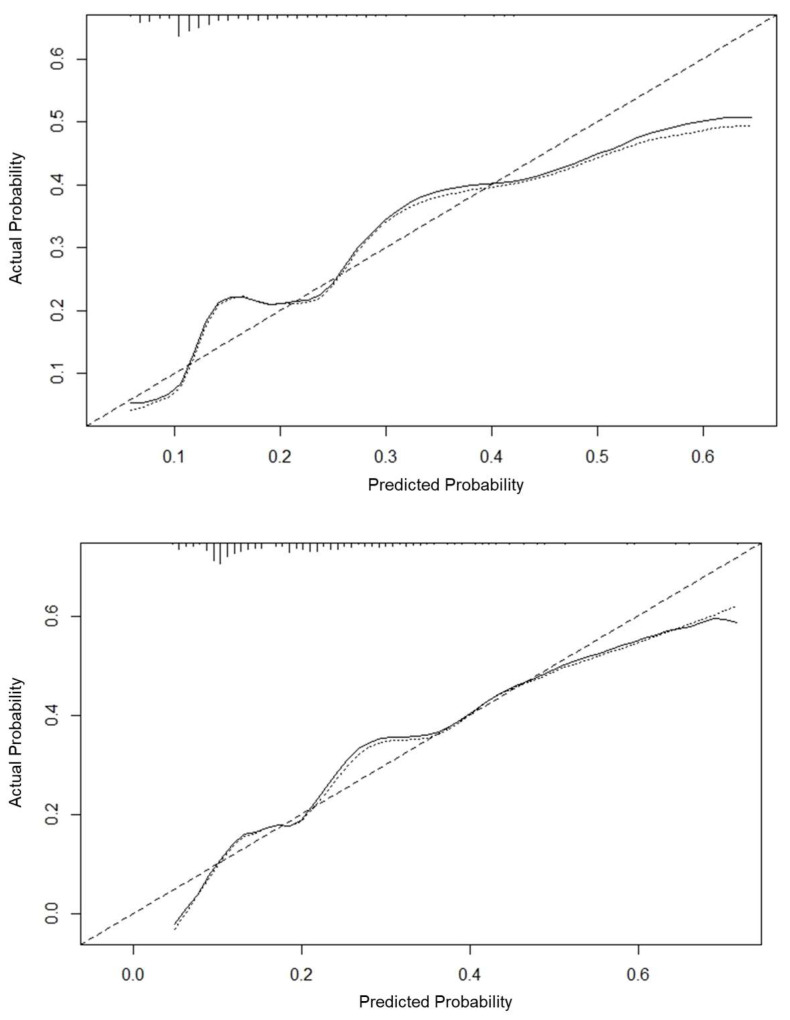
Correction curve of nomogram. The 45° dashed line represents the ideal curve, the dotted line represents the standard curve, and the solid line represents the calibration curve. (above: derivation set, below: verification set).

The internal and external validation of the nomogram model was carried out. The area under the curve (AUC) of the receiver operating characteristic (ROC) curve of the derivation set was 0.705 (sensitivity 0.789, specificity 0.582), the AUC of the ROC curve of the verification set was 0.733 (sensitivity 0.892, specificity 0.489), and the AUC was greater than 0.7 (see Figure 3). The nomogram correction curve was made, the consistency index (C-index) generated by the derivation set was 0.706, the C-index generated by the verification set was 0.733, and the calibration curve was close to the standard curve, indicating that the predicted probability was consistent with the actual probability, and the nomogram had a good fitting effect (see Figure 4).

### 3.6. Yodon’s Index

A large number of guidelines have confirmed that voriconazole-related liver injury was related to the blood concentration. The Yoden’s index of the blood concentration was calculated to provide reference for the prediction of voriconazole-related liver injury. The results showed that the Yodon’s index was 0.343, and the corresponding blood concentration was 4.375 mg/L (sensitivity 0.5855, specificity 0.7576), indicating that the liver injury was prone to occur when the blood concentration was greater than 4.375 mg/L after the use of voriconazole.

## 4. Discussion

The incidence of liver injury (transaminase > 3 ULN) recorded in the instructions of voriconazole was 18.04% (319/1768) in adults and 25.80% (73/283) in children. In this study (CIOMS standard), the incidence of voriconazole-related liver injury in hospitalized patients was 15.82% (217/1372). The incidence of liver injury in transplant patients was higher, which was more likely to lead to premature drug withdrawal. In a single center retrospective study on the use of voriconazole to prevent fungal infection after lung transplantation by Samanta P. et al. in 2021, 35.76% (54/151) of patients stopped taking the drug due to adverse reactions, especially liver injury (18.54%, 28/151) [24]. In 2021, Bogler Y. et al. conducted a study on the use of voriconazole to prevent fungal infection after allogeneic hematopoietic cell transplantation, wherein 22.86% (48/210) of patients stopped taking voriconazole too early due to liver injury [8]. Early withdrawal may increase the risk of fungal recurrence. In 2020, Chan S.Y. et al. reported that voriconazole was used for antifungal prevention in allogeneic hematopoietic cell transplantation recipients. The median use time of voriconazole in the standard group was 90 days (n = 180) and the median use time of voriconazole in the early withdrawal group was 20 days (n = 147), and on the 180th day after HCT, 5.4% (8/147) of patients in the early withdrawal group and 2.8% (5/180) of patients in the standard group had invasive fungal infections (log rank test: *p* = 0.13), 15.6% (23/147) and 7.8% (14/180) patients died (log rank test: *p* = 0.03) [25].

The influence of voriconazole on liver function ranges from mild abnormal liver biochemical indicators to fatal fulminant liver failure, which increases the cost of handling adverse reactions. There is no report on the disposal cost of voriconazole-related liver injury, and the report on the treatment cost of liver injury caused by other drugs is extremely rare. In 2019, Yan-ping Zhao et al. reported that 386 patients with drug-induced liver injury had an average cost of 785.92 Chinese yuan/case [26]. This study found that the disposal cost of voriconazole-related liver injury (cost of liver protecting drugs, solvents, infusion sets) was higher than that of non-liver injury (median 101.90 vs. 0.00 Chinese yuan, *p* < 0.001), and the cost of treatment of severe liver injury was higher than that of general liver injury (median 993.59 vs. 0.00 Chinese yuan, *p* < 0.001). In Table 2, patients without liver injury incurred disposal cost, because few patients used hepatoprotective drugs when they had abnormal liver biochemical indicators, although they did not meet the criteria for liver injury. The median disposal cost of the general liver injury group was 0.00 (0.00, 410.48) yuan. This is because most patients with mild liver injury discontinued or reduced the dose according to the voriconazole blood concentration, rather than adding hepatoprotective drugs. However, a small number of patients used many hepatoprotective drugs, so the per capita cost was 483.23 yuan. The disposal cost of severe voriconazole-related liver injury was 1082.58 yuan on mean and 993.59 (361.70, 1451.76) yuan on median, which was similar.

Identifying and intervening in the cases of patients at high risk of voriconazole-related liver injury can help reduce the incidence of adverse reactions and the cost of treatment. Voriconazole-related liver injury may be associated with the treatment duration of different fungal diseases, such as an anti-candida treatment duration of ≥2 weeks, an anti-aspergillus treatment duration of ≥6 weeks, and an anti-cryptococcus treatment duration of ≥6 months, as long treatment duration may increase the risk of liver injury. This study found that voriconazole-related liver injury was not associate with the fungal disease type. It may be that these patients received TDM, which facilitated dosing adjustment or drug replacement. Invasive fungal disease was divided into “proven”, “probable”, and “possible”. The probable and possible invasive fungal diseases also require the same antifungal therapy as proven invasive fungal diseases, although the pathogen is not known. In this study, nearly half of the patients on voriconazole had no microbiological evidence (culture negative or histopathology not performed), which were mostly probable or possible invasive fungal diseases.

The practice guide for drug-induced liver injury issued by the European Society for Liver Research in 2019 pointed out that host factors such as old age, chronic liver disease, diabetes, tumors, and heart disease may be risk factors for drug-induced liver injury caused by some drugs, but the evidence was limited [27]. This study found that voriconazole-related liver injury was not related to age, chronic liver disease (chronic hepatitis B, decompensated cirrhosis) with normal liver biochemical indicators, diabetes, tumors, or heart disease.

In 2012, Luong M.L. et al. found that voriconazole-related liver injury was not significantly related to the history of liver disease through multiple factor logistic regression analysis [28]. In 2015, Lo Re V 3rd et al. investigated the incidence of liver injury in outpatients after taking azole antifungal drugs, and found that the incidence of voriconazole liver injury in outpatients with chronic liver disease was 4.12% (4/97), similar to that in outpatients without chronic liver disease (4.46%, 17/381) (*p* = 1.000) [29]. It should be noted that only patients with chronic liver disease with normal liver biochemistry before using voriconazole were included in this study according to the nanofiltration standard. If patients with liver dysfunction with obvious abnormal liver biochemistry were encountered in the actual clinical practice, the addition of voriconazole should be cautious and dynamically monitored for liver function.

In addition to the above-mentioned comorbidities, we also analyzed the correlation between voriconazole-related liver injury and other common comorbidities such as bacterial infection, hypertension, hyperlipidemia, and chronic renal failure, since concomitant medications for comorbidities may increase the risk of liver injury. No association was found in this study. In particular, about a third of the patients had coexisting bacterial infections. It should be noted that severe infections with systemic inflammation (n = 64, see Figure 1) were excluded from this study. In clinical practice, we need to pay attention to the superimposed risk of liver injury from voriconazole and severe bacterial infections and their antibacterial drugs.

A large number of studies have confirmed that voriconazole-related liver injury was related to serum trough concentration. In 2016, Jin H. and other meta-analyses showed that the incidence of liver injury was significantly increased when the serum trough concentration was >3.0, >4.0, >5.5, and >6.0 mg/L [30]. In 2016, Wang Y. et al. reported that the risk of liver injury in patients in intensive care units (*n* = 63) was significantly increased when the serum trough concentration of voriconazole was >4.0 mg/L [31]. In 2019, Hirata A. et al. reported that the risk of liver injury increased in patients with hypoproteinemia (*n* = 42) when the serum trough concentration was >4.2 mg/L [32]. The critical value of liver injury reported by Hamada Y. et al. in 2020 was 3.5 mg/L [33]. In this study, the critical value was calculated by the Yoden’s index. It was found that after the use of voriconazole, the trough concentration exceeded 4.375 mg/L, which likely led to liver injury, similar to the literature report.

This study found that voriconazole-related liver injury was associated with hypoproteinemia, and the proportion of patients with hypoproteinemia was 24.78% (340/1372). The protein binding rate of voriconazole was 58%. Hypoalbuminemia can increase the concentration of plasma-free voriconazole, and more free molecules are distributed to liver and other tissues and organs, increasing the risk of adverse reactions.

Most studies show that the incidence of liver injury in transplant patients after using voriconazole is high. Pablo Solís-Muñoz et al. found that patients with liver diseases had poor tolerance to voriconazole treatment after liver transplantation [34]. Adding voriconazole at the early stage of lung transplantation (within 30 days after transplantation) increased the risk of liver injury (OR 4.37, 95% CI: 1.53–12.43, *p* = 0.006) [28]. However, another study showed that receiving T cell transplantation was a protective factor against voriconazole-related liver injury [25]. In this study, voriconazole liver injury was negatively related to the transplant status, which may be because doctors paid more attention to transplant patients, used drugs more cautiously, and monitored the blood concentration more promptly and frequently, which prevented some drug-induced liver injury as early as possible and thus promoted a reduction in the actual incidence of liver injury. A multicenter study on voriconazole TDM in Japan (*n* = 401) found that voriconazole-related liver injury was not related to the initial serum trough concentration (ROC curve AUC = 0.562, critical value 3.6 mg/L, OR 1.67, *p* = 0.292), but related to the serum trough concentration at the time of adverse reaction (ROC curve AUC = 0.725, critical value 3.5 mg/L, OR 5.20, *p* < 0.001), which indicated that adjusting the administration scheme promptly based on the blood concentration monitoring results can reduce the incidence of liver injury [33]. Similarly, the risk of liver injury in transplant patients can be reduced by monitoring the blood–drug concentration more frequently and adjusting the dosage more promptly.

The association between voriconazole-related liver injury and *CYP2C19* and *UGT1A4* gene polymorphisms is unclear [11,12]. Since *CYP2C19* polymorphism affects the serum trough concentration of voriconazole [35], it is still necessary to further explore the correlation between *CYP2C19* polymorphism and liver injury. In this study, few patients were detected for the *CYP2C19* gene, so the gene polymorphism was not included in the risk factor analysis.

In 2021, Hanai Y. et al. established a quantitative relationship between liver injury (P) and serum trough concentration (*X*, range 1–6 mg/L): P = 0.1198 × e^0.2298*X*^ (*p* = 0.007) [14]. This study established a quantitative relationship between liver injury (P) and multiple risk factors: Logit(P)= −2.219 + 0.094 × *C*_min_ + 0.544 × *Hypoproteinemia* − 0.589 × *Transplantation*. The prediction model integrated more risk factors and had a wider range of trough concentrations, which can provide help for the accurate and safe clinical use of drugs with a narrow therapeutic window and promote the drug safety of patients.

The nomogram transforms the complex regression equation into a simple and visual graph, which makes the results of the prediction model more readable and is gradually used in medical and adverse drug reaction prediction research. In 2020, Xu N. et al. developed a nomogram to predict vancomycin related nephrotoxicity in hospitalized patients [36]. In 2021, Yu C. et al. developed a nomogram to predict drug-induced acute renal injury in hospitalized patients [37]. In this study, a predictive model of voriconazole-related liver injury with a nomogram was constructed, and the model was verified internally and externally. The results showed that the C-index of the derivation set and verification set were 0.706 and 0.733. The calibration curves were close to the standard curves, which showed that the model had good prediction accuracy. The AUC of ROC curve of the derivation set and verification set was 0.705 and 0.733, respectively, which indicate that the model has good discrimination.

This study was the first to establish a quantitative relationship between voriconazole-related liver injury and multiple risk factors, providing a scientific patient screening method for targeted pharmaceutical services such as drug monitoring and drug intervention for high-risk patients in the future. It has been suggested that providing pharmaceutical services for voriconazole blood concentration can improve the rate of reaching the standard of blood concentration and reduce the incidence of adverse reactions [27].

### Limitations

This study also has several limitations. First, the retrospective study has limitations on the estimation of incidence and cost. This retrospective study investigated the frequency of new cases of liver injury during voriconazole treatment in hospitalized patients who met the inclusion and exclusion criteria. Adverse reactions were considered new cases only if they occurred after the administration of voriconazole, so as to increase the statistical accuracy of the incidence. The disposal cost of adverse reactions (liver injury) included the cost of hepatoprotective drugs, solvents, and infusion sets, but the cost of medical staff time and the labor loss caused by patients’ prolonged hospitalization were not available, so the cost was underestimated. Increased biochemical testing due to adverse reactions was also excluded, since retrospective study cannot accurately distinguish whether biochemical tests were performed because of adverse effects or the disease itself. Second, the duration of voriconazole use was not considered, because a few patients were already using voriconazole before admission, and the initial time of using voriconazole was unknown. Long term medication may increase the cumulative incidence of voriconazole-related liver injury [38]. Third, in terms of drug factors, only the dosage and blood concentration of voriconazole were included, lacking information on combinations with other drugs, which reduced the prediction efficiency of the model to a certain extent. However, Luong M.L. et al. reported that voriconazole combined with other drugs with known hepatotoxicity (such as statins, calcium channel blockers, quinolones, antipsychotics, azathioprine, and allopurinol) did not increase the risk of liver injury [28]. Fourth, as with other single center studies, the results and conclusions should be carefully extrapolated to other medical institutions. The medical institution in this study has 4300 beds, and it is a national center for difficult, critical, and severe diseases in Western China. The proportion of patients with difficult and complex diseases receiving treatment is more than 80%. Therefore, the model can still be optimized by including more variables such as medication duration, drug combination, and multi-center research.

## 5. Conclusions

Voriconazole-related liver injury was common and increased the disposal cost of adverse drug reactions. The prediction model was convenient for application, with good accuracy and differentiation, to predict patients at high risk of voriconazole-related liver injury based on patient and drug factors initially. The prediction model will be helpful to develop clinical individualized medication of voriconazole and promote medication safety, so as to reduce the incidence of voriconazole-related liver injuries and the cost of treatment. We can develop an early warning system for liver injury and provide targeted pharmaceutical services such as drug monitoring and drug intervention for high-risk patients.

## Figures and Tables

**Table 1:** Baseline characteristics and clinical data in the derivation and validation cohorts.

Variables	Total (*n* = 1372)	Groups			
		Derivation Set (*n* = 1035)	Validation Set (*n* = 337)	Statistical Value	*p*
Basic information					
Age (y)	48.55 ± 17.14	48.19 ± 17.40	49.66 ± 16.29	−1.410 ^a^	0.159
Male (*n*, %)	939 (68.44)	715 (69.08)	224 (66.47)	0.804 ^b^	0.370
Height (cm)	164.82 ± 7.10	164.92 ± 7.14	164.52 ± 6.96	0.911 ^a^	0.363
Weight (kg)	59.76 ± 10.99	58.89 ± 10.35	62.45 ± 12.39	−4.761 ^a^	<0.001
BMI (kg/m^2^)	17.66 ± 3.51	17.93 ± 3.61	16.84 ± 3.04	5.437 ^a^	<0.001
Fungal disease type (*n*, %)					
Aspergillosis	539 (39.29)	443 (42.80)	96 (28.49)	21.842 ^b^	<0.001
Cryptococcosis	97 (7.07)	80 (7.73)	17 (5.04)	2.789 ^b^	0.095
Candidiasis	52 (3.79)	35 (3.38)	17 (5.04)	1.928 ^b^	0.165
Talaromycosis	4 (0.29)	1 (0.10)	3 (0.89)		0.048 ^c^
Unknown pathogen	680 (49.56)	476 (45.99)	204 (60.53)	128.199 ^b^	<0.001
Major comorbidities (*n*, %)					
Bacterial infection	492 (35.86)	402 (38.84)	90 (26.71)	16.275 ^b^	<0.001
Chronic viral hepatitis B (normal liver biochemistry)	91 (6.63)	73 (7.05)	18 (5.34)	1.203 ^b^	0.273
Decompensated cirrhosis (normal liver biochemistry)	60 (4.37)	42 (4.06)	18 (5.34)	1.101 ^b^	0.317
Chronic renal failure	213 (15.52)	166 (16.04)	47 (13.95)	0.848 ^b^	0.357
Abnormal renal function	41 (2.99)	30 (2.90)	11 (3.26)	0.117 ^b^	0.732
Cancer	246 (17.93)	211 (20.39)	35 (10.39)	17.279 ^b^	<0.001
Renal transplantation	138 (10.06)	120 (11.59)	18 (5.34)	10.988 ^b^	0.001
Stem cell transplantation	75 (5.47)	62 (5.99)	13 (3.86)	2.238 ^b^	0.135
Lung transplantation	29 (2.11)	23 (2.22)	6 (1.78)	0.240 ^b^	0.624
Liver transplantation	5 (0.36)	4 (0.39)	1 (0.30)	0.400 ^c^	1.000
Autoimmune disease	93 (6.78)	67 (6.47)	26 (7.72)	0.620 ^d^	0.431
Diabetes	314 (22.89)	227 (21.93)	87 (25.82)	2.173 ^b^	0.140
Chronic obstructive pulmonary disease	51 (3.72)	49 (4.73)	2 (0.59)	12.179 ^b^	<0.001
Asthma	22 (1.60)	12 (1.16)	10 (2.97)	5.267 ^b^	0.022
Hypertension	487 (35.50)	337 (32.56)	110 (32.64)	0.001 ^b^	0.978
Heart disease	166 (12.10)	122 (11.79)	44 (13.06)	0.385 ^b^	0.535
Hyperlipidemia	40 (2.92)	28 (2.71)	12 (3.56)	0.657 ^b^	0.417
Anemia	327 (23.83)	226 (21.84)	101 (29.97)	9.267 ^b^	0.002
Hypoproteinemia	340 (24.78)	192 (18.55)	148 (43.92)	87.756 ^b^	<0.001
Voriconazole dose (mg/kg)	3.31 ± 0.51	3.34 ± 0.48	3.22 ± 0.58	3.573 ^a^	<0.001
Voriconazole trough concentration (mg/L)					
*C*_min_, M (P_25_, P_75_)	2.66 (1.20,5.00)	2.62 (1.25,5.16)	2.62 (1.10,4.54)	2.472 ^e^	0.013
*C*_min_ < 1.0 mg/L (*n*, %)	279 (20.34)	227 (21.93)	52 (15.43)	6.634 ^b^	0.010
*C*_min_ [1.0, 5.5] mg/L (*n*, %)	797 (58.09)	596 (57.58)	201 (59.64)	0.725 ^b^	0.394
*C*_min_ > 5.5mg/L (*n*, %)	296 (21.57)	212 (20.48)	84 (24.93)	2.966 ^b^	0.085
Liver biochemical indicator					
ALT [M(P_25_,P_75_), IU/L]	22 (12,39)	22 (12,39)	23 (12,39)	0.947 ^e^	0.344
AST [M(P_25_,P_75_), IU/L]	24 (17,40)	24 (17,40)	24 (18,40)	2.832 ^e^	0.005
TBil [M(P_25_,P_75_), μmol/L]	7.7 (5.3,11.8)	7.9 (5.4,12.1)	7.4 (5.2,11.2)	4.047 ^e^	<0.001
DBil [M(P_25_,P_75_), μmol/L]	3.5 (2.3,6.2)	3.5 (2.3,6.3)	3.4 (2.1,5.6)	4.442 ^e^	<0.001
ALP [M(P_25_,P_75_), IU/L]	99 (72,144)	99 (72,146)	101 (72,138)	5.553 ^e^	<0.001

Note ^a^: *t*-value, ^b^: χ^2^-value, ^c^: Fisher’s value, ^d^: continuous correctionχ^2^-value, ^e^: Z-value.

**Table 2:** Disposal cost of voriconazole-related liver injury.

Group	*n*	Disposal Cost (Chinese Yuan)		
		Range	Mean	Median (P_25_, P_75_)
non VCZ-LI	1155	0.00~4823.43	58.19	0.00 (0.00, 0.00)
VCZ-LI	217	0.00~8372.65	599.23	101.90 (0.00, 786.48)
General VCZ-LI	175	0.00~4540.50	483.23	0.00 (0.00, 410.48)
Severe VCZ-LI	42	0.00~8372.65	1082.58	993.59 (361.70, 1451.76)

Note: VCZ-LI, voriconazole-related liver injury; 1 Chinese Yuan = 0.1400 US dollar.

**Table 3:** Sensitivity analysis of disposal cost based on the discount rate between 0 and 8%.

Group	Discount Rate of 0%		Discount Rate of 8%	
	Mean	Median (P_25_, P_75_)	Mean	Median (P_25_, P_75_)
non VCZ-LI (*n* = 1155)	46.06	0.00 (0.00, 0.00)	66.64	0.00 (0.00, 0.00)
VCZ-LI (*n* = 217)	466.29	79.84 (0.00, 617.40)	693.05	117.31 (0.00, 900.29)
General VCZ-LI (*n* = 175)	373.48	0.00 (0.00, 321.62)	561.04	0.00 (0.00, 472.57)
Severe VCZ-LI (*n* = 42)	853.02	770.00 (294.08, 1124.14)	1243.09	1128.31 (409.63, 1689.02)

Note: VCZ-LI, voriconazole-related liver injury; 1 Chinese Yuan = 0.1400 US dollar.

**Table 4:** Single factor analysis of voriconazole-related liver injury.

Variables	VCZ-LI Group (*n* = 152)	Non VCZ-LI Group (*n* = 883)	Statistical Value	*p*
Basic information				
Age (y)	49.55 ± 15.90	47.96 ± 17.64	1.117 ^a^	0.265
Male (*n*, %)	99 (65.13)	616 (69.76)	1.302 ^b^	0.254
Height (㎝)	164.25 ± 7.18	165.04 ± 7.14	−1.255 ^a^	0.210
Weight (kg)	58.54 ± 9.56	58.95 ± 10.48	−0.445 ^a^	0.657
BMI (kg/m^2^)	17.74 ± 3.43	17.96 ± 3.64	−0.679 ^a^	0.497
Fungal disease (*n*, %)				
Aspergillosis	67 (44.08)	376 (42.58)	0.119 ^b^	0.730
Cryptococcosis	4 (2.63)	76 (8.61)	6.492 ^b^	0.011
Candidiasis	5 (3.29)	30 (3.40)	0.005 ^b^	0.946
Unknown pathogen	76 (50.00)	400 (45.30)	1.153 ^b^	0.283
Major comorbidities (*n*, %)				
Bacterial infection	62 (40.79)	340 (38.51)	0.285 ^b^	0.594
Chronic viral hepatitis B (normal liver biochemistry)	6 (3.95)	67 (7.59)	2.621 ^b^	0.105
Decompensated cirrhosis (normal liver biochemistry)	10 (6.58)	32 (3.62)	2.908 ^b^	0.088
Chronic renal failure	21 (13.82)	145 (16.42)	0.654 ^b^	0.419
Abnormal renal function	4 (2.63)	26 (2.94)	0.045 ^b^	0.832
Cancer	38 (25.00)	173 (19.59)	2.336 ^b^	0.126
Transplantation	19 (12.50)	190 (21.52)	6.543 ^b^	0.011
Autoimmune disease	12 (7.89)	55 (6.23)	0.594 ^b^	0.441
Diabetes	32 (21.05)	195 (22.08)	0.081 ^b^	0.777
Chronic obstructive pulmonary disease or Asthma	10 (6.58)	51 (5.78)	0.151 ^b^	0.698
Hypertension	47 (30.92)	330 (37.37)	2.331 ^b^	0.127
Heart disease	26 (17.11)	96 (10.87)	4.846 ^b^	0.028
Hyperlipidemia	4 (2.63)	24 (2.72)	0.004 ^b^	0.952
Anemia	41 (26.97)	185 (20.95)	2.756 ^b^	0.097
Hypoproteinemia	47 (30.92)	145 (16.42)	18.044 ^b^	<0.001
Voriconazole dose (mg/kg)	3.36 ± 0.46	3.34 ± 0.48	0.448 ^a^	0.654
*C*_min_, [M (P_25_, P_75_), mg/L]	4.96 (2.78, 7.46)	2.33 (1.01, 4.34)	−8.317 ^c^	<0.001

Note: VCZ-LI, voriconazole-related liver injury; ^a^: *t*-value, ^b^: χ^2^-value, ^c^: Z-value.

**Table 5:** Predictors included in the multivariable logistic regression model.

Predictors	β	S.E	Wald χ^2^	*p*	OR	95%CI
*C*_min_ (mg/L)	0.094	0.019	23.940	<0.001	1.099	(1.058, 1.140)
hypoproteinemia	0.544	0.217	6.275	0.012	1.723	(1.126, 2.636)
transplantation	−0.589	0.273	4.645	0.031	0.555	(0.325, 0.948)
Constant	−2.219	0.160	192.567	<0.001		

Abbreviations: OR, odds ratio; CI, confidence intervention.

## Data Availability

The datasets used and/or analyzed during the current study are available from the corresponding author on reasonable request.
